# Community structure of gut bacteria of *Dendroctonus armandi* (Coleoptera: Curculionidae: Scolytinae) larvae during overwintering stage

**DOI:** 10.1038/s41598-017-14724-y

**Published:** 2017-10-27

**Authors:** Juan Wang, Hui Chen, Ming Tang

**Affiliations:** 1 State Key Laboratory for Conservation and Utilization of Subtropical Agro-bioresources (South China Agricultural University), Guangdong Key Laboratory for Innovative Development and Utilization of Forest Plant Germplasm, College of Forestry and Landscape Architecture, South China Agricultural University, Guangzhou, 510642 China; 2 College of Forestry, Henan Agricultural University, Zhengzhou, 450002 China

## Abstract

Survival rate at low temperature becomes a crucial strategy since temperature change often leads to fluctuations in the insect population. Microbes play important roles in the process of resisting low temperature. In this study, we analyzed gut bacterial communities from Chinese white pine beetle *Dendroctonus armandi* which remained overwintering process under natural conditions from October 2015 to January 2016, monthly, in the Qinling Mountains, Shaanxi, China using Illumina MiSeq sequencing. A total of 835,227 high-quality sequences and 48 singleton operational taxonomic units were obtained. Gut bacterial communities showed variation in relative abundance during the overwintering stage. As ambient temperature declined, Proteobacteria (mostly γ-proteobacteria) became the predominant phylum in the larvae guts, and followed with Actinobacteria and Firmicutes, respectively. In particular, there was no Deinococcus-Thermus in January 2016. Thermoleophilia appeared in November and December 2015, but not for October 2015 and January 2016, nor did δ-proteobacteria. By contrast, gut bacterial community compositions increased in relative abundance in November and December 2015. This study provided initial evidence that gut bacterial communities were associated with the larvae overwintering process at low temperature. Moreover, no complementary studies combining overwintering process of Coleoptera insect and high-throughput sequencing were carried out, paying particular attention to insect in cold season.

## Introduction

Bark beetle, especially *Dendroctonus* specie which causes serious damage to coniferous trees has been widely recognized for their ecological significance in forests^1,2^. Their damage results in enormous economic loss and becomes a dominating disruptive factor for the ecosystem^3^. Microbial communities, ranging from simple to complex, can be harbored by bark beetles^4^. Microbes make important contributions to the host insects’ life history and physiology^5,6^.

The Chinese white pine beetle *Dendroctonus armandi* Tsai and Li (Coleoptera: Curculionidae: Scolytinae) is the most damaging forest insect in the Qinling Mountains, Shaanxi, China^7,8^. *D. armandi* as a leading serious forest pest invades the phloem of *Pinus armandi* Franch and causes high mortality of healthy *P. armandi* in natural forest ecosystems, reaching epidemic proportions^7,8^. Various aspects of *D. armandi*’s biology and physiology have been studied because of its importance, such as its ecological niche^9^, life cycle^10^ and symbiotic fungi^11^. However, little is known about its larvae gut bacterial communities during overwintering stage at low temperature.

Insect faces a great challenge in surviving at low temperatures in frigid and temperate zones^8,12,13^. Insect survival rate in overwintering stage is a major factor limiting their population and distribution^8^. Hence, the lowest temperature in winter is considered as an vital element on insects’ range expansion and outbreak frequency^8,14–16^. The insects’ survival rates at low temperatures influence their population dynamics^8,17^ and geographical distribution^8,18^.

Gut microbiota play important roles in insects’ life histories contributing to effective reproduction rate, community interactions and survival rates by metabolizing toxins and providing protection against a variety of environmental stresses^19–22^. Bacteria can contribute to the insects which feed on a nutrient-poor food source by nutritional supplements^23–26^. Insects that exploited woody substrates are particularly nutrient poor and available to competitors once the woody substrate are depleted^27–29^. Bacteria can provide nutritional supplements such as amino acids^23^, essential vitamins^24^, nitrogen and carbon compounds^25,26^. Such relationships seem to be widespread among insects that exploit woody substrates. Host insects show slow growth and high mortality if they are short of symbiont^30^. Most of studies focus on the variation of endosymbionts during the insect development stage^31–33^; however, it is unknown if the composition of endosymbionts varies during the overwintering stage at low ambient temperature. A certain type of bacteria was first reported in stinkbugs (Plataspidae) via a “symbiont capsule” which was transmitted vertically^34^. We hypothesized that some relationships existed between gut bacterial community structure and the overwintering process of *D. armandi* larvae at low temperature. In order to understanding the roles of endosymbiont community members in bark beetles, the community composition should be defined in the first step^35,36^.

In our study, gut bacterial communities of *D. armandi* larvae at low temperatures were investigated using high-throughput sequencing method during overwintering stage. We quantified the composition and structure of gut bacterial communities in each month from October 2015 to January 2016 in winter.

## Results

### Environmental conditions

The average of daily total sunshine duration started with 5.285 ± 1.417 (s.d.) hours in October 2015 and declined to 3.218 ± 1.137 (s.d.), 3.837 ± 0.820 (s.d.) hours in November and December 2015, respectively. Finally, the value was up to 4.069 ± 0.818 (s.d.) hours in January 2016 (Table 1). The average daily rainfall was 1.016 ± 3.108 (s.d.) mm in October 2015. In December 2015 and January 2016, the value decreased to 0.023 ± 0.062 (s.d.) and 0.048 ± 0.146 (s.d.) mm, before it increased to 1.740 ± 2.416 (s.d.) mm in November 2015 (Table 1). The mean daily air temperature was around 0 °C, and ranged from −5.045 ± 2.314 (s.d.) to 5.069 ± 1.958 (s.d.) °C in December 2015. The mean daily maximum air humidity was 96.155 ± 3.593 (s.d.)% in October 2015 and the mean daily minimum air humidity was 34.326 ± 3.502 (s.d.)% in December 2015 (Table 1). The mean daily maximum and minimum air humidity were decreased during the winter and ranged from the lowest value of 34.643 ± 3.620 (s.d.)% to the highest value of 81.670 ± 5.986 (s.d.)% in January 2016 (Table 1).

**Table 1:** Fundamental meteorological data from October 2015 to January 2016.

Sampling Time	SunDur (Hour)	Rain (mm)	AirT_Max (°C)	AirT_Min (°C)	RH_Max (%)	RH_Min (%)
Oct. 2015	5.285 ± 1.417	1.016 ± 0.108	17.285 ± 1.073	6.026 ± 1.350	96.155 ± 3.593	47.837 ± 3.200
Nov. 2015	3.218 ± 1.137	1.740 ± 0.416	9.118 ± 1.410	0.089 ± 0.426	93.492 ± 8.524	52.563 ± 5.032
Dec. 2015	3.837 ± 0.820	0.023 ± 0.062	5.069 ± 1.958	−5.045 ± 1.314	83.035 ± 9.095	34.326 ± 3.502
Jan. 2016	4.069 ± 0.818	0.048 ± 0.146	5.711 ± 1.044	−5.167 ± 1.365	81.670 ± 5.986	34.643 ± 3.620

SunDur, accumulated duration of sunshine; Rain, average rainfall; AirT_Max, maximum air temperature; AirT_Min, minimum air temperature; RH_Max, maximum relative humidity; RH_Min, minimum relative humidity.

### Overview of sequencing analysis

**Figure 1:**
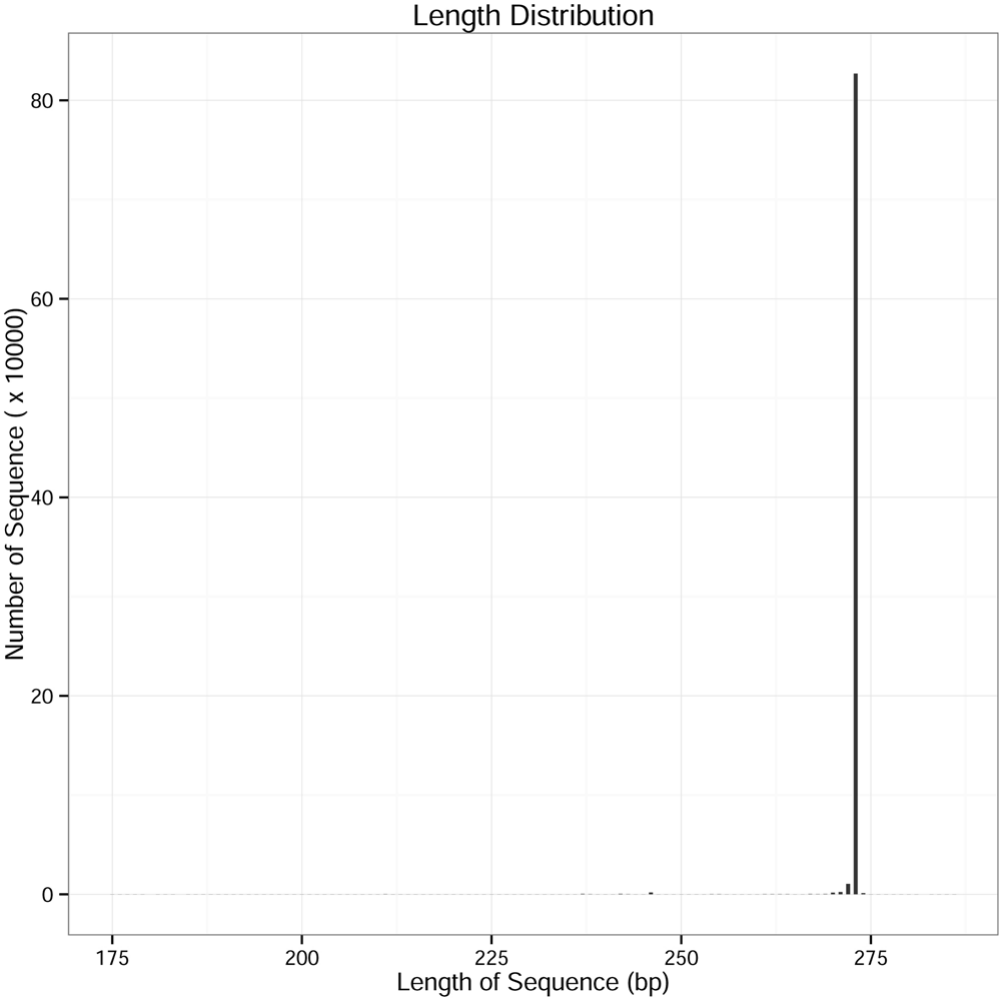
The length distribution of the high quality sequences.

**Figure 2:**
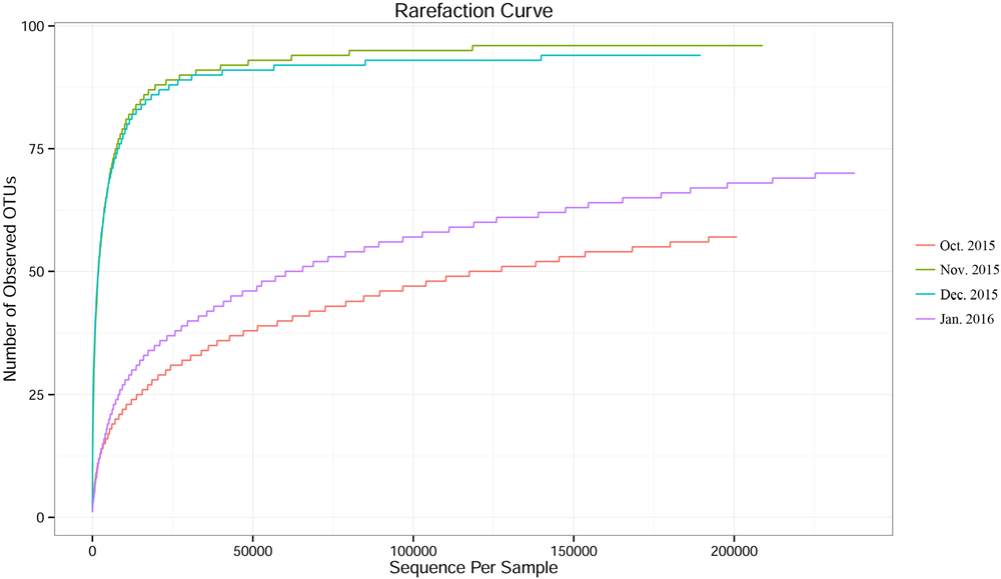
The rarefaction curve in each month from October 2015 to January 2016.

**Figure 3:**
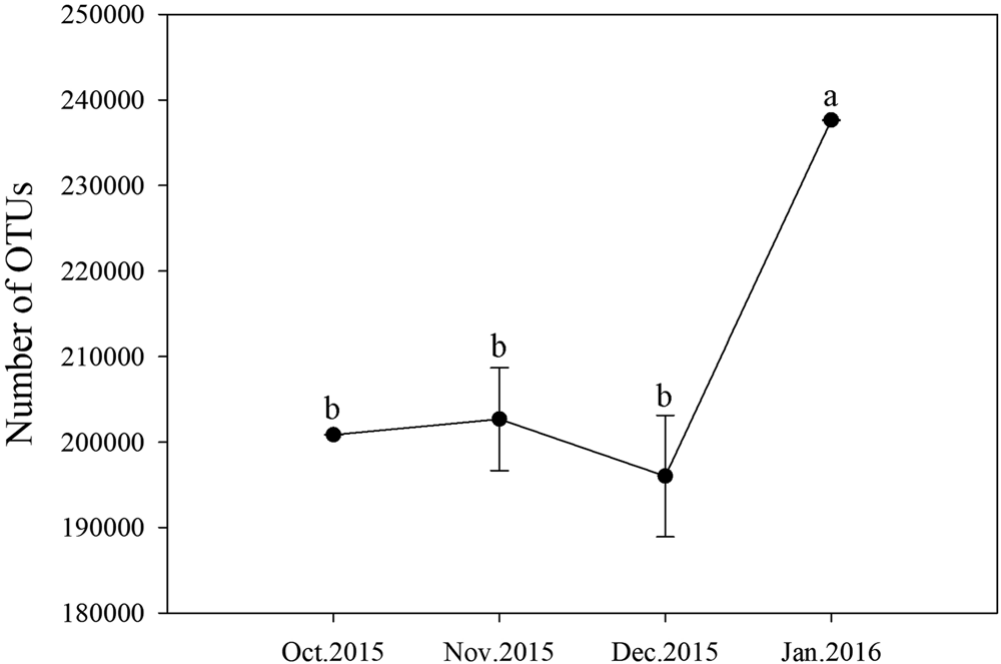
Number of OTUs in *Dendroctonus armandi* larval gut bacteria in each month from October 2015 to January 2016. Note: Values are presented as means ± SE. Values with the different lowercase letters in the contents are significantly different (P < 0.05, Tukey’s multiple comparisons test after analysis of variance).

The proportion which equaled the number of high quality sequences/valid sequences (Shared reads/Total reads) was over 90% in each month, expect for December 2015 (89.57%) in the current study (Table 2). After sequence trimming, quality filtering and removal of chimeras, 835,227 high-quality sequences remained, with an average length of 273 bases (Fig. 1). The mean number of sequences per sample was 208,807 ± 9,698 (s.d.). The rarefaction curves in four months had approached the plateau phase, and they were unlikely that more bacterial communities composition would be detected with additional sequencing efforts (Fig. 2). These high-quality sequences were clustered into different operational taxonomic units (OTUs) by the UPARSE pipeline using a threshold of 97% identity (200,833; 208,496; 188,307 and 237,592 OTUs for October, November, December 2015 and January 2016, respectively). Number of OTUs dramatically increased in January 2016 compared with October, November and December 2015 (*F* = 16.808, d.f. = 3, *P* < 0.001) (Fig. 3). These high-quality sequences were classified into 7 phyla, 15 classes, 28 oredrs, 54 families and 64 genera (Supplementary Tables S1, S2 and S3). In total, 835,227 OTUs were singletons. We classified 566,638 OTUs of high-quality sequences to the Proteobacteria, 232,256 OTUs to the Firmicutes, 22,727 OTUs to the Actinobacteria, 10,491 OTUs to the Tenericutes, 1,613 OTUs to the Bacteroidetes, 1,355 OTUs to the Acidobacteria and 147 OTUs to the Deinococcus-Thermus on the phylum level (Table 2). Detailed characteristics of each phylum were listed in Supplementary Tables S1, S2 and S3.

**Table 2:** Shared seven phyla communities of *Dendroctonus armandi* larvae from October 2015 to January 2016.

Phylum	Oct. 2015	Nov. 2015	Dec. 2015	Jan. 2016	SUM	Shared OTUs
Proteobacteria	24,443	147,705	158,997	235,494	566,638	25
Actinobacteria	706	18,450	3,368	203	22,727	9
Bacteroidetes	78	575	918	42	1,613	4
Firmicutes	175,477	33,023	21,980	1,776	232,256	7
Acidobacteria	6	422	904	23	1,355	2
Tenericutes	122	8,319	1,996	54	10,491	1
Deinococcus-Thermus	1	2	144	0	147	0
Total shared reads	200,833	208,496	188,307	237,592	835,277	48
Total reads	200,912	209,647	210,228	237,721		
Shared reads/Total reads	99.96%	99.45%	89.57%	99.95%		

OTU,operational taxonomic unit.

### Bacterial diversity during overwintering period

**Figure 4:**
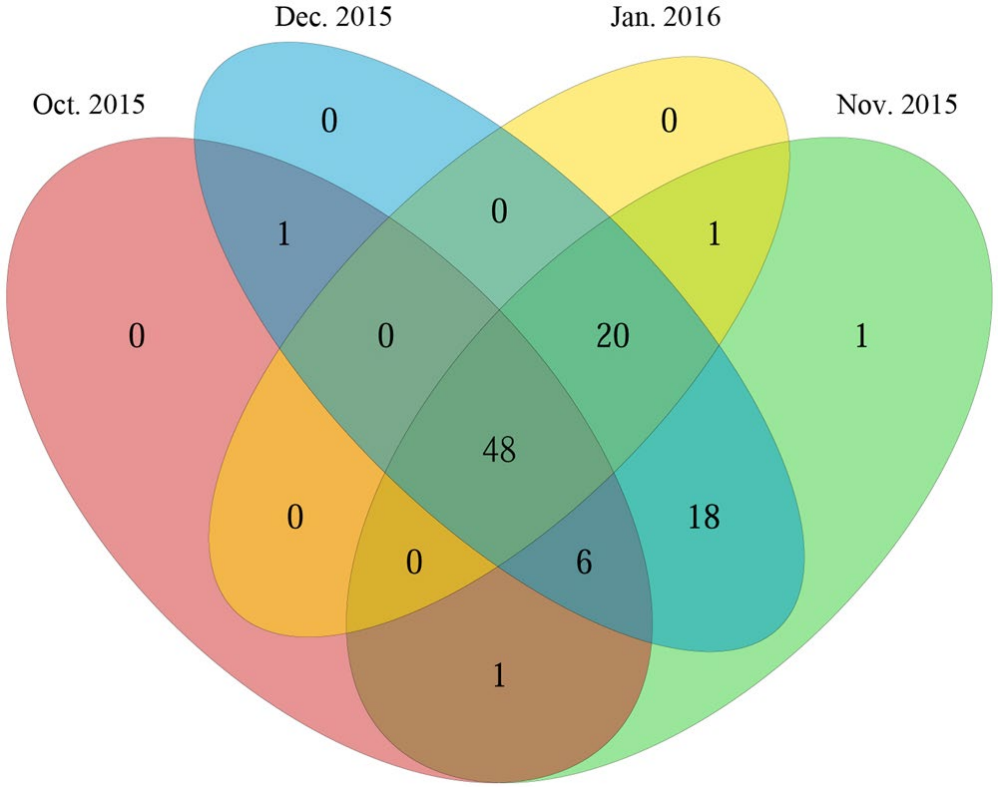
Shared operational taxonomic unit (OTU) analysis of the different communities.

The community richness and community diversity for *D. armandi* larvae rapidly increased in November and December 2015 compared with the larvae collected in October 2015 and January 2016 (Table 3). A Venn diagram was used to compare the similarities and differences between the communities in the different months (Fig. 4). The communities among Oct. 2015, Nov. 2015, Dec. 2015 and Jan. 2016 had 48 OTUs in common altogether (Fig. 4), with the common OTUs comprising 99.96%, 99.45%, 89.57% and 99.95% of the sequences in the October, November, December 2015 and January 2016 communities, respectively (Table 2).

**Table 3:** Biodiversity index values of *Dendroctonus armandi* larvae from October 2015 to January 2016.

Sampling time	Chao	ACE	Shannon	Simpson
Oct. 2015	67.11111	70.6169	0.642307	0.225086
Nov. 2015	96	96	2.313493	0.569439
Dec. 2015	94.5	95.27586	2.286132	0.523645
Jan. 2016	83.125	80.75568	0.135518	0.023940

### Bacterial community composition and structure succession analysis

**Figure 5:**
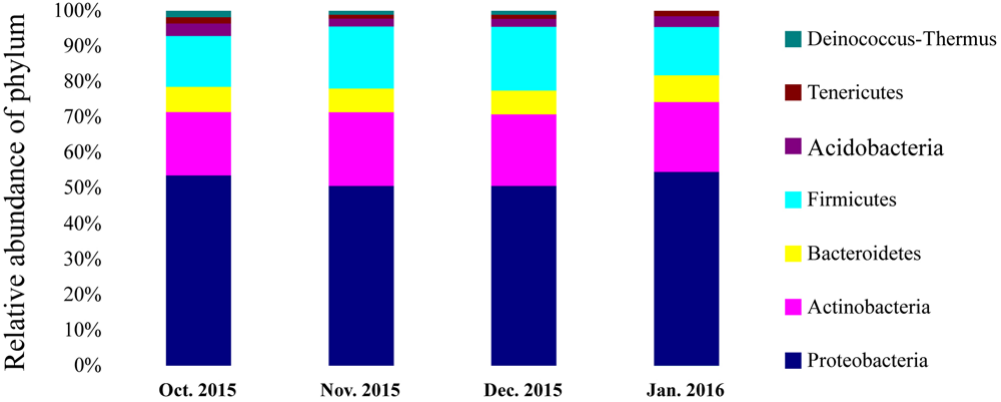
Bacterial community structure variation during overwintering stage at the phylum level of *Dendroctonus armandi* larvae.

**Figure 6:**
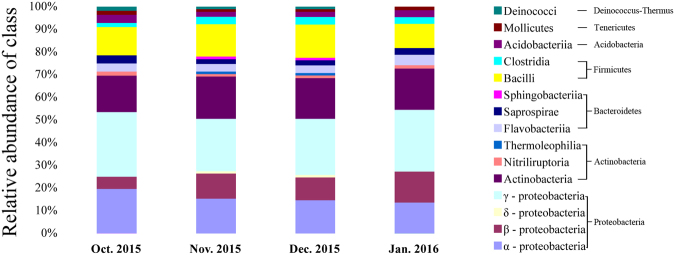
Bacterial community structure variation during overwintering stage at the class level of *Dendroctonus armandi* larvae.

**Figure 7:**
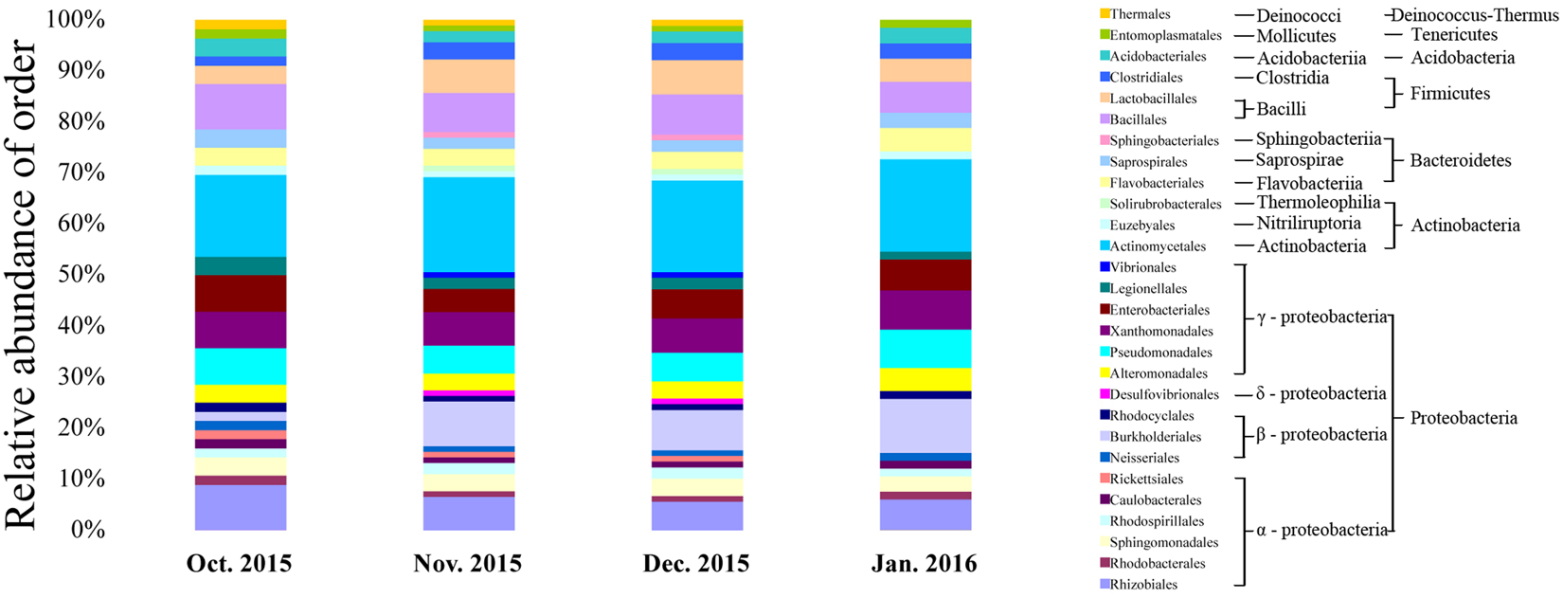
Bacterial community structure variation during overwintering stage at the order level of *Dendroctonus armandi* larvae.

**Figure 8:**
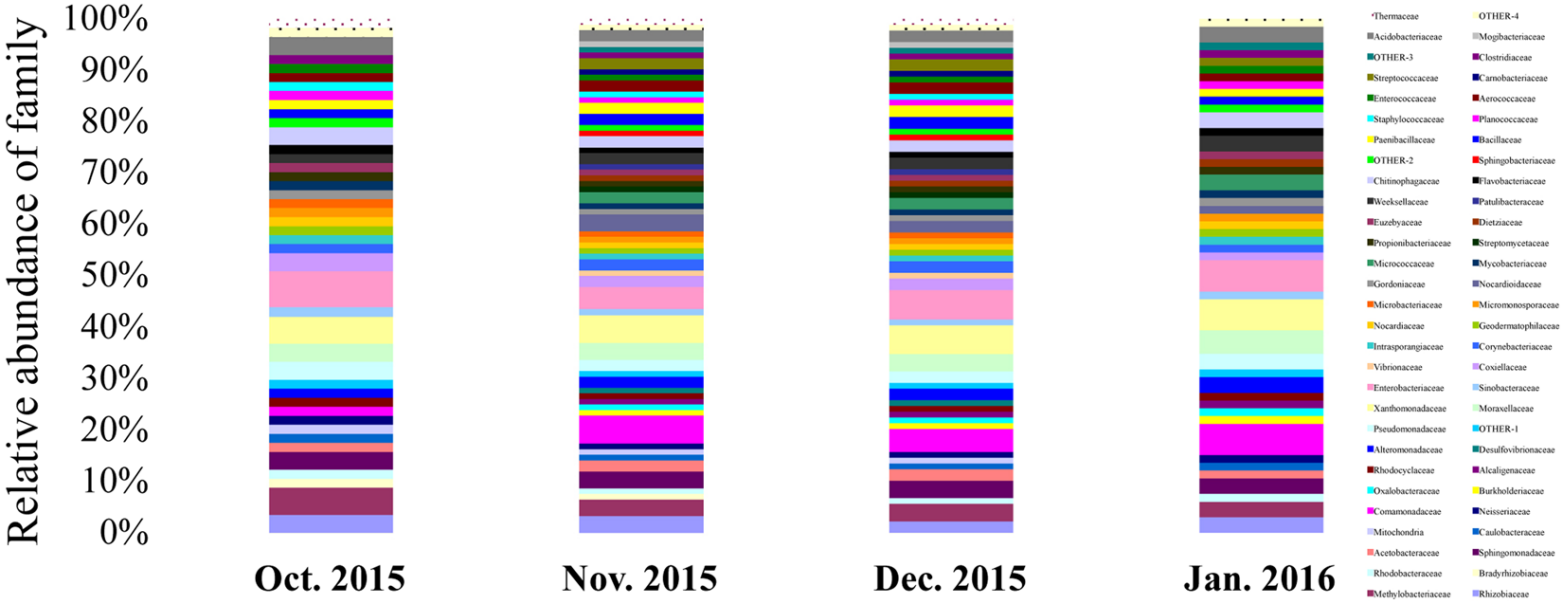
Bacterial community structure variation during overwintering stage at the family level of *Dendroctonus armandi* larvae.

Gradually decreasing temperatures in winter resulted in a complicated metabolic process and also the enhancement of survival rate at low temperature^13^. To identify gut bacterial community structure succession at low temperature during overwintering stage, the 16 S ribosomal ribonucleic acid (rRNA) sequences were classified at the phylum, the class, the order and the family levels with quantitative polymerase chain reaction (qPCR) amplifcations. There were obvious trends and changes in the relative abundance of the different bacterial taxa in each month from October 2015 to January 2016 (Figs 5–8). During the overwintering stage at low ambient temperature, Proteobacteria was the dominant phylum at 51.99 ± 9.31 (s.d.)% and followed with Actinobacteria and Firmicutes at 19.87 ± 5.60 (s.d.)% and 16.28 ± 4.37 (s.d.)%, respectively (Fig. 5). There was no Deinococcus-Thermus in January 2016. However, Deinococcus-Thermus was contained in October, November and December 2015 (Fig. 5). At the class level, γ-proteobacteria (49.04 ± 8.52 (s.d.)%) was the dominant class compared with α-proteobacteria, β-proteobacteria and δ-proteobacteria (29.94 ± 8.91 (s.d.)%, 19.75 ± 6.15 (s.d.)% and 1.27 ± 0.84 (s.d.)%, respectively) (Fig. 6). Actinobacteria (90.00 ± 7.46 (s.d.)%) was the predominant of the Actinobacteria. Furthermore, Bacilli (81.63 ± 8.13 (s.d.)%) was ascendant of the Firmicutes. Thermoleophilia appeared in November and December 2015, but not for October 2015 and January 2016, nor did δ-proteobacteria (Fig. 6). Gut bacterial community compositions increased in relative abundance in November and December 2015 (Figs 7 and 8). At the class level, Desulfovibrionales, Vibrionales, Solirubrobacterales and Sphingobacteriales were specific in November and December 2015 (Fig. 7).

There were obvious differences between bacterial community richness and community diversity. Chao and ACE index values which represented the bacterial community richness increased prior from 67.111 and 70.617 in October 2015 to both 96 in November 2015, slightly declined in December 2015 from 96 to 94.5 and 95.276, respectively, and finally decreased to 83.125 and 80.756. (Table 3). Shannon and Simpson index values which represented the bacterial community diversity had the same trends compared with Chao and ACE index values. The values increased prior from October 2015 to November 2015, slightly declined in December 2015 and finally decreased in January 2016 (Table 3).

### Clustering patterns of samples during overwintering stage

**Figure 9:**
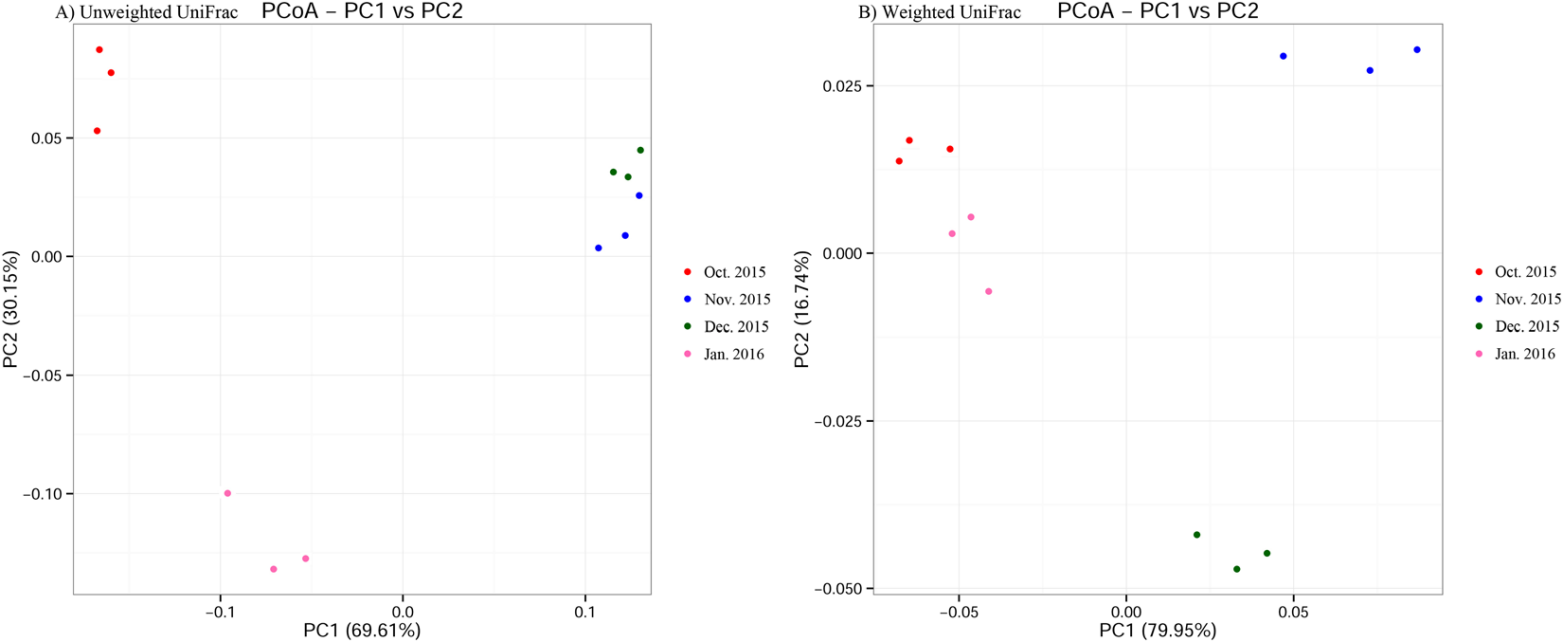
Two-dimensional principal coordinates analysis (PCoA) plot of unweighted (**A**) and weighted (**B**) unifrac distance matrices for *Dendroctonus armandi* larvae in each month from October 2015 to January 2016.

**Figure 10:**
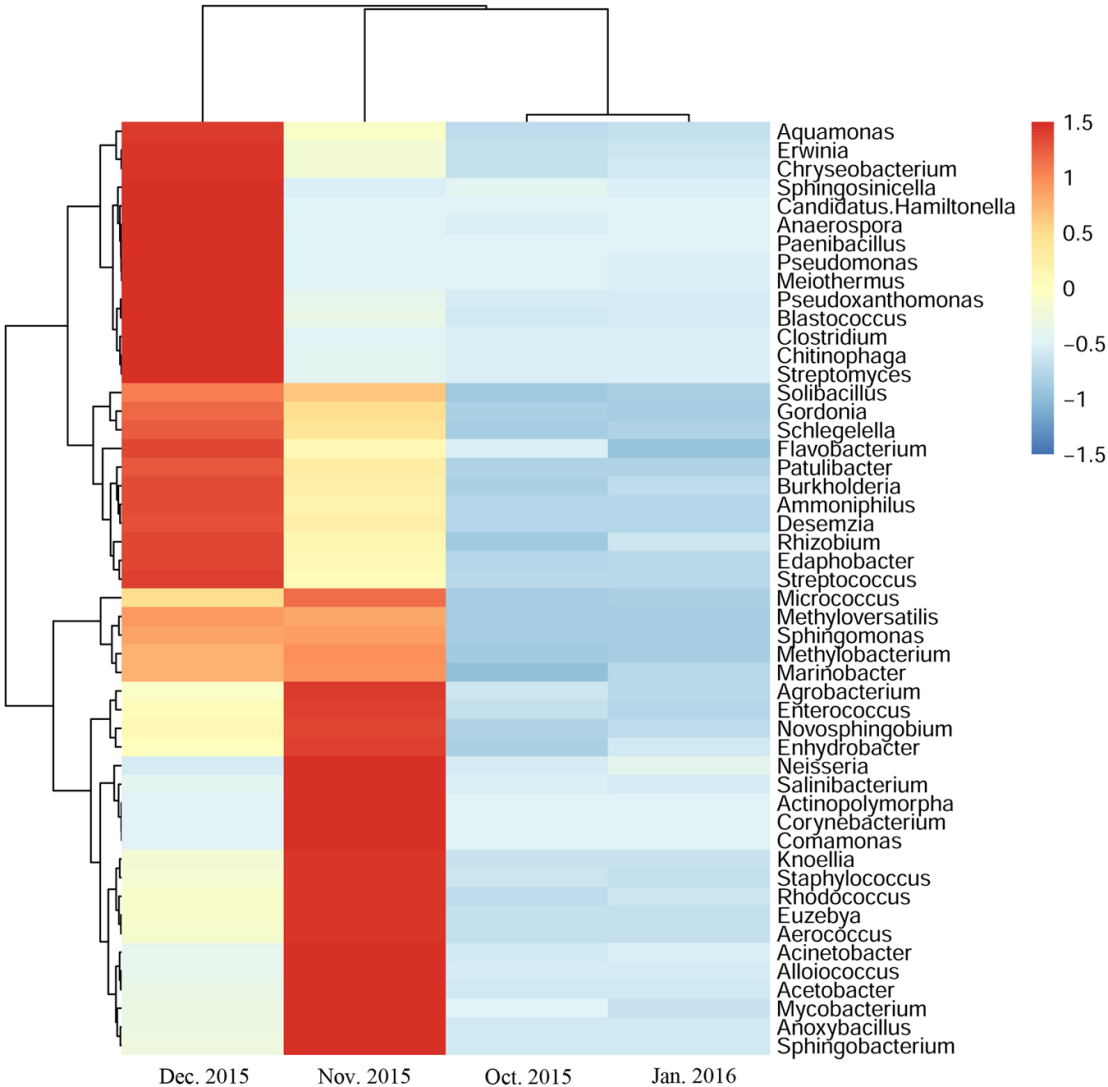
Heat maps of *Dendroctonus armandi* larval gut bacterial family in each month from October 2015 to January 2016.

Principal coordinate analysis (PCoA) showed a distinct clustering of gut bacterial communities every month from overwintering larvae at low temperature (Fig. 9) PCoA revealed the overwintering pattern in four months for the unweighted and weighted unifrac distances (Fig. 9). According to the unweighted unifrac PCoA, larvae collected from November and December 2015 formed a unique cluster, separated from the other two months (Fig. 9). According to principal coordinate 1 (PC1) and PC2 analysis (69.61% and 30.15% of variance explained, respectively), the differences of gut bacterial communities at low temperature during overwintering stage were great between November, December 2015 and October 2015, January 2016, while the differences were little in November 2015 compared with December 2015 (Fig. 9). According to weighted unifrac PCoA, the larvae collected in November and December were separated and the gut bacterial communities (collected in October 2015 and January 2016) were clustered based on PC1 and PC2 analysis (79.95% and 16.74% of variance explained, respectively) (Fig. 9). At the genus level, the larvae collected in November and December 2015 generally also had more OTUs than those in October 2015 and January 2016, when the relative abundances of the top 50 OTUs were compared with Z-score method (Fig. 10). This analysis revealed similar results with Fig. 8. The larvae collected in November, December 2015 and: October 2015, January 2016 formed distinct clusters, while the larvae collected in October 2015 and January 2016 were more similar, so were the larvae collected in November and December 2015.

### Differences between samples among four months in the overwintering larvae

Differences in the gut bacterial communities composition among four months were tested using the Tukey’s tests for multiple comparisons (Supplementary Tables S4 and S5). For the overwintering larvae at low temperature, the genera *Pseudomonas*, *Pseudoxanthomonas*, *Aquamonas*, *Anaerospora*, *Sphingosinicella*, *Brevundimonas* and *Steroidobacter* which belonged to the Proteobacteria were more prevalent in December 2015, and *Rhizobium*, *Burkholderia*, *Schlegelella*, *Enhydrobacter*, *Cellvibrio*, *Janthinobacterium* and *Achromobacter* (Proteobacteria) were only absent in October 2015. *Acetobacter*, *Sphingomonas*, *Stenotrophomonas* and *Photobacterium* (Proteobacteria) only appeared in November and December 2015. Furthermore, the genera *Corynebacterium*, *Knoellia*, *Rhodococcus*, *Euzebya*, *Mycobacterium* and *Propionibacterium* which belonged to the Actinobacteria, *Alloiococcus* and *Anoxybacillus* which belonged to the Firmicutes were more important in November 2015. The genera *Chryseobacterium*, *Chitinophaga* and *Flavobacterium* belonged to the Bacteroidetes, *Edaphobacter* from Acidobacteria were enriched in December 2015. The genera *Patulibacter*, *Streptomyces* and *Pimelobacter* belonged to the Actinobacteria, *Paenibacillus, Aerococcus, Desemzia, Streptococcus* and *Bacillus* belonged to the Firmicutes, *Sphingobacterium* from Acidobacteria were absent in Octoer 2015 and January 2016. The genus *Meiothermus* from Deinococcus-Thermus appeared in October, November and December 2015, and was merely absent in January 2016.

## Discussion

This study analyzed the gut bacterial communities structure and relative diversity with low temprature in each month from October 2015 to January 2016 during overwintering stage of *D. armandi* larvae. Low temprature is a leading factor of winter survival for most insects in frigid and temperate zones^13,17,18^. Insects survived at low temperature can keep vital bodily functions well and provides tolerance to low temperatures in adverse low ambient environment^37^. Freezing-tolerant insects can survive after the formation of internal ice, whereas most insect are freeze susceptible (freezing-intolerant) species which cannot tolerate the internal ice formation. These insects improved their supercooling capacity by increasing the contents of polyols or other forms of cryoprotectants^8,38^. Whether the gut bacterial communities composition and structure is vital for insects during winter is still unknown. In our study, the dominant phyla during overwintering stage were Proteobacteria, Firmicutes and Actinobacteria (Supplementary Tables S1 and S2), and the dominant classes were γ-proteobacteria, Actinobacteria, Bacilli and α-proteobacteria (Supplementary Tables S1, S2 and S3). The changes of gut bacterial relative diversity accorded with the OTU richness estimation on the whole. The special phyla was Deinococcus-Thermus which just existed in October, November and December 2015. Furthermore, the unique families were Desulfovibrionaceae, Vibrionaceae, Streptomycetaceae, Patulibacteraceae, Sphingobacteriaceae, Carnobacteriaceae and Mogibacteriaceae in November and December 2015. We hypothesized these families were associated with the insect overwintering process and resisting to low temperature.

The present results illuminated that gut bacterial communities differed during each month from October 2015 to January 2016. While, there were still some obvious similarities of insect communities as the result of similar environmental variables such as temperature^36^. So far, few studies analyzed the diversity of gut bacterial species from overwintering insects. Mostly, researchers coped with the gut bacterial communities composition in insects’ development stages^33^. Even further, there was no complementary researches combining overwintering process of Coleoptera insect and high-throughput sequencing to our knowledge, especially for insect in cold season.

Gut bacterial communities composition and diversity showed differences at low temperatures of Chinese white pine beetle larvae. The phyla of Proteobacteria, Actinobacteria, Bacteroidetes, Firmicutes, Acidobacteria and Tenericutes appeared in each month from October 2015 to January 2016, when the mean monthly ambient temperatures declined from 11.0 °C to 0.5 °C (Table 1). Meanwhile, gut bacterial communities from *D. armandi* larvae could be monitored immediately, annotating into 7 phyla, 15 classes, 28 orders, 54 families, 64 genera. Their appearances were possibly in correlation with overwintering process and environmental low temperature changes. Among these identified bacteria, γ-proteobacteria from Proteobacteria and Bacilli from Firmicutes exhibited higher presence at low ambient temperature during overwintering period.

The Proteobacteria was the most dominant, accounting for 67.84%, which was similar to the dominant bacteria for some insects such as *Bactrocera minax* (Diptera: Tephritidae)^39^, *Ceratitis capitata* (Diptera: Tephritidae)^40^, *Lutzomyia longipalpis* (Diptera: Psychodidae)^41^, *Schistocerca gregaria* (Orthoptera: Acrididae)^42^, *Acyrthosiphon pisum* (Hemiptera: Aphididae)^43^, *Anoplophora glabripennis* (Coleoptera: Cerambycidae)^44^, *Riptortus clavatus* (Hemiptera: Alydidae)^45^ and *Saperda vestita* (Coleoptera: Cerambycidae)^44^. However, the results were different with some other insects about dominant bacterium. The predominant bacteria of *Drosophila* species^46^, *Lymantria dispar* (Lepidoptera: Lymantriidae)^35^, *Helicoverpa armigera* (Lepidoptera: Noctuidae)^47^, *Bombyx mori* (Lepidoptera: Bombycidae)^48^ and *Melolontha melolontha* (Coleoptera: Scarabaeidae)^49^ was Firmicutes. Furthermore, Firmicutes and Bacteroidetes were the predominant bacterium of *Musca domestica* (Diptera: Muscidae)^50^, Firmicutes, Bacteroidetes and Spirochaetae were the predominant bacterium of *Coptotermes formosanus* (Isoptera: Rhinotermitidae)^51,52^, Proteobacteria and Actinobacteria were the predominant bacterium of *Holotrichia parallela* (Coleoptera: Scarabaeidae)^53^, Proteobacteria, Firmicutes, Actinobacteria and Bacteroidetes were the predominant bacterium of *Anoplophora glabripennis* (Coleoptera: Cerambycidae)^44^.

Environment changes including altitude, latitude, and ambient temperature influences insects’ survival rate in frigid and temperature zones because they are poikilothermic animals^54,55^. According to Bryant *et al*.^56^ and Singh *et al*.^57^, bacterial diversity was significantly correlated with elevation. This was not surprising that the larvae overwintering process was a temperature dependent biochemical process. This result supported our previous hypothesis that low temperature had an effect on larvae gut bacterial communities diversity on the Qinling Mountains due to the high altitude. Lastly, we attempted to identify the environmental variables which controlled larvae gut bacterial communities in order to understand their survival rates in winter. Low ambient temperature was previously shown to be the first priority predictor of variation in bacterial diversity. Therefore, studying the relationship between low ambient temperature and bacterial diversity during overwintering stage of Chinese white pine beetle larvae was the next purpose.

## Materials and Method

### Study sites

Overwintering *D. armandi* larvae were collected in the Huoditang Experimental Forest Station which located on the southern slope of the middle Qinling Mountains (33°17′–33°27′ N, 108°22′–108°40′ E) of Northwest Agricultural and Forestry University, Shaanxi, China.

### Insect collection and dissection

Chinese white pine beetle (*D. armandi*) larvae were collected from host trees Chinese white pines (*P. armandi*) in each month from October 2015 to January 2016. The overwintering larvae were collected from three sample plots, 20 m × 20 m in each one, from the above site in each month. Five trees were selected by five-point sampling method in each sampling plot. The phloem, 20 cm × 20 cm, was peeled off from four directions. All overwintering *D. armandi* larvae were gathered into glass culture dishes with sterile moist paper using fine forceps and directly transported to the laboratory^33^. 150 larvae samples in each month were gathered for high-throughput sequencing analysis from attacked Chinese white pine (*P. armandi*).

The overwintering larvae were treated as following:(1)

rinsed with sterile water once; (2) surface sterilized for 3 min with 70% ethanol; (3) rinsed with sterile water twice; (4) dissected under a stereomicroscope using insect pins; (5) gathered the larval gut into 10 mM sterilized phosphate-buffered saline (Sangon Company, Shanghai, pH 7.4).

150 larvae guts in a month were transferred into three 1.5-mL microcentrifuge tubes (fifty larval guts in each tubes). These three 1.5-mL microcentrifuge tubes were homogenized several times before grinding in liquid nitrogen, and then vortexed at the speed of 2500 r/min for 3 min with 500 mL Tris-EDTA (Sangon Company, Shanghai, pH 8.0). The microbial cells were separated from gut wall tissues after centrifuging at the speed of 4000 r/min for 15 s. The supernatants contained gut bacteria and fungi were totally transferred into three new 1.5-mL microcentrifuge tubes for gut bacterial DNA extraction. All of the steps above were accomplished in a sterile environment with biological air clean bench (Suzhou Antai Airtech, Jiangsu, China).

### Bacterial DNA extraction

The E.Z.N.A. Bacterial DNA Kit (Omega Biotech, USA) was used to extract overwintering *D. armandi* larval gut bacterial deoxyribonucleic acid (DNA) following the instruction booklet. The gut bacterial DNA was stored at −20 °C before using. DNA samples were mixed in equal concentrations, and the mixed DNA specimens were sent to JBYH Biotechnology Co.,Ltd (Wuhan, China) for analysis by MiSeq sequencing.

### Bioinformatics and statistical analysis

Paired-end reads were truncated by cutting off the barcode and primer sequence based on their unique barcodes. Then, the single, longer sequences were merged by these paired-end reads using FLASH (Version 1.2.7)^58^. High-quality clean tags performed by quality filtering on the raw tags under specific filtering conditions by QIIME (Version 1.7.0) quality controlled process^59,60^. Chimeric sequences were detected and removed using the UCHIME algorithm^61^.

Clustering was performed using the UPARSE pipeline (version 7.0.1001)^62^, and similar sequences were assigned to OTUs using the threshold of 97% identity. A typical sequence was picked by selecting the longest sequence that had the largest number of hits to other sequences in the OTU. The RDP classifier (version 2.2)^63^ was used to annotate taxonomic information for each representative sequence. The typical sequences were aligned using the Greengenes database^64^, with a minimum identity of 80%. The differences in the dominant species were conducted using MUSCLE (Version 3.8.31) in order to study the phylogenetic relationships of different OTUs of different samples, multiple sequence alignments^65^.

The alpha diversity analysis included observed species, Ace and Chao estimators, Simpson and Shannon diversity indices estimate of coverage. Rarefaction curves were generated based on observed species. The beta diversity among overwintering *D. armandi* larval gut bacterial communities was evaluated using both weighted and unweighted unifrac distances^66^. UPGMA was used to accomplish the hierarchical clustering of samples. The Z-score method was used for heat maps. PCoA was performed to make the differences in larval gut bacterial community composition and structure concrete on the unifrac distances of the unweighted and weighted distance matrices, respectively. Statistical analyses of numbers of OTUs (expressed as the mean ± SE) in each month from October 2015 to January 2016 in *D. armandi* larval gut bacteria were performed using ANOVA followed by Tukey’s tests for multiple comparisons to detect significant differences performed with SPSS 18.0 (IBM SPSS Statistics, Chicago, IL, USA) and Sigma Plot 12.5 sofware (Systat Sofware Inc., San Jose, CA, USA).

### Ethics approval and consent to participate

Our manuscript report data collected from insect named Chinese white pine beetle. The larvae Chinese white pine beetles were collected in the Huoditang Experimental Forest Station of Northwest A&F University. Huoditang Experimental Forest Station agreed us to collect insects for experiments, in order to find a way of controlling the damage.

### Availability of data and materials

The datasets supporting the conclusions of this article included within the article (and its additional files) are available in the repository.

## Electronic supplementary material

Supplementary Tables
