# Supplementary material for: Community structure of gut bacteria of Dendroctonus armandi (Coleoptera: Curculionidae: Scolytinae) larvae during overwintering stage
Source: Sci Rep. 2017 Oct 27;7:14242. doi: 10.1038/s41598-017-14724-y (PMC5660234; doi:10.1038/s41598-017-14724-y)
Supplement: Supplementary file 1 — Supplementary Tables [file 41598_2017_14724_MOESM1_ESM.pdf]

Community structure of gut bacteria of *Dendroctonus armandi* (Coleoptera: Curculionidae: Scolytinae) larvae during overwintering stage

Juan Wang[2], Hui Chen[1]* & Ming Tang[1]

Supplementary Table S1

The number of operational taxonomic units (OTUs) of Actinobacteria in each month from October 2015 to January 2016 of *Dendroctonus armandi* larvae.

**Phylum**	**Class**	**Order**	**Family**	**Oct. 2015 (OTUs)**	**Nov. 2015 (OTUs)**	**Dec. 2015 (OTUs)**	**Jan. 2016 (OTUs)**
			Corynebacteriaceae	33	11,704	79	81
			Intrasporangiaceae	13	3,454	669	11
			Geodermatophilaceae	16	210	1,661	37
			Nocardiaceae	2	1,154	297	56
			Micromonosporaceae	622	479	119	2
			Microbacteriaceae	3	240	11	0
	Actinobacteria	Actinomycetales	Nocardioidaceae	0	310	43	1
Actinobacteria			Gordoniaceae	4	87	118	2
			Mycobacteriaceae	7	96	14	1
			Micrococcaceae	0	95	62	4
			Streptomycetaceae	0	4	64	0
			Propionibacteriaceae	3	42	9	1
			Dietziaceae	0	18	28	4
	Nitriliruptoria	Euzebyales	Euzebyaceae	3	518	126	3
	Thermoleophilia	Solirubrobacterales	Patulibacteraceae	0	39	68	0
**Total shared reads**				**706**	**18,450**	**3,368**	**203**

Supplementary Table S2

The number of operational taxonomic units (OTUs) of Proteobacteria in each month from October 2015 to January 2016 of *Dendroctonus armandi* larvae.

Phylum	Class	Order	Family	Oct. 2015 (OTUs)	Nov. 2015 (OTUs)	Dec. 2015 (OTUs)	Jan. 2016 (OTUs)
Proteobacteria	α - proteobacteria	Rhizobiales	Rhizobiaceae	276	4,294	1,290	86
			Methylobacteriaceae	5	1,089	846	20
			Bradyrhizobiaceae	1	44	0	0
		Rhodobacterales	Rhodobacteraceae	7	42	1,803	12
		Sphingomonadales	Sphingomonadaceae	77	98	1,430	9
		Rhodospirillales	Acetobacteraceae	337	567	182	1
		Caulobacterales	Caulobacteraceae	1	4	46	4
		Rickettsiales	Mitochondria	1	22	23	0
	β - proteobacteria	Neisseriales	Neisseriaceae	3	691	4	64
		Burkholderiales	Comamonadaceae	0	467	149	10
			Burkholderiaceae	0	40	75	5
			Oxalobacteraceae	0	42	1	1
			Alcaligenaceae	0	4	37	1
		Rhodocyclales	Rhodocyclaceae	1	101	96	1
	δ - proteobacteria	Desulfovibrionales	Desulfovibrionaceae	0	23	223	0
	γ - proteobacteria	Alteromonadales	Alteromonadaceae	6	450	386	75
			UNCLASSFIED	23,470	134,696	129,727	234,741
		Pseudomonadales	Pseudomonadaceae	94	194	7,783	47
			Moraxellaceae	26	1,418	442	62
		Xanthomonadales	Xanthomonadaceae	32	1,008	8,316	110
			Sinobacteraceae	1	13	27	1
		Enterobacteriales	Enterobacteriaceae	102	2,300	5,733	216
		Legionellales	Coxiellaceae	3	45	377	28
		Vibrionales	Vibrionaceae	0	53	1	0
**Total shared reads**				**24,443**	**147,705**	**158,997**	**235,494**

Supplementary Table S3

The number of operational taxonomic units (OTUs) of Bacteroidetes, Firmicutes, Acidobacteria, Tenericutes and Deinococcus-Thermus in each month from October 2015 to January 2016 of *Dendroctonus armandi* larvae.

**Phylum**	**Class**	**Order**	**Family**	**Oct. 2015 (OTUs)**	**Nov. 2015 (OTUs)**	**Dec. 2015 (OTUs)**	**Jan. 2016 (OTUs)**
Bacteroidetes	Flavobacteriia	Flavobacteriales	Weeksellaceae	5	131	512	29
			Flavobacteriaceae	21	48	92	5
	Saprospirae	Saprospirales	Chitinophagaceae	52	214	290	8
	Sphingobacteriia	Sphingobacteriales	Sphingobacteriaceae	0	182	24	0
Firmicutes	Bacilli	Bacillales	UNCLASSFIED	175,226	3,672	9,279	1,451
			Bacillaceae	52	12,259	1,502	105
			Paenibacillaceae	5	452	1,268	6
			Planococcaceae	4	367	429	16
			Staphylococcaceae	2	71	15	0
		Lactobacillales	Aerococcaceae	106	15,577	1,411	101
			Enterococcaceae	10	111	41	6
			Carnobacteriaceae	0	26	49	0
			Streptococcaceae	0	43	60	1
	Clostridia	Clostridiales	Clostridiaceae	72	340	7,631	89
			UNCLASSFIED	0	60	268	1
			Mogibacteriaceae	0	45	27	0
Acidobacteria	Acidobacteriia	Acidobacteriales	Acidobacteriaceae	6	422	904	23
Tenericutes	Mollicutes	Entomoplasmatales	UNCLASSFIED	122	8,319	1,996	54
Deinococcus-Thermus	Deinococci	Thermales	Thermaceae	1	2	144	0
**Total shared reads**				**175,684**	**42,341**	**25,942**	**1,895**

Supplementary Table S4

Genera with significantly different abundances in each month from October 2015 to January 2016 of *Dendroctonus armandi* larvae of Proteobacteria.

Phylum	Genus	mean abundance				*p* value
		**Oct. 2015**	**Nov. 2015**	**Dec. 2015**	**Jan. 2016**	
Proteobacteria	*Pseudomonas*	0.000463069	0.000698862	0.040858536	0.00018518	0.0308
	Pseudoxanthomonas	0.000149377	0.004068718	0.043311947	0.000248309	0.0251
	Agrobacterium	0.00136929	0.020372312	0.006399975	0.000315647	0.0314
	Aquamonas	0.0000996	0.006572177	0.022682186	0.000366151	0.0158
	Anaerospora	0.0000349	0.000201043	0.009512905	0.0000505	0.0106
	Sphingosinicella	0.000373443	0.0000574	0.007286369	0.0000168	0.0082
	Erwinia	0.0000299	0.001282843	0.005666589	0.000210432	0.0241
	Methylobacterium	0.0000199	0.004949476	0.004400312	0.0000842	0.0311
	Marinobacter	0.0000299	0.00207744	0.001888863	0.000265144	0.0225
	Neisseria	0.0000149	0.003307629	0.0000211	0.000269352	0.0120
	Comamonas	0.00000498	0.001498246	0.0000106	0.00000842	0.0106
	Methyloversatilis	0.00000498	0.000483459	0.000506511	0.00000421	0.0073
	Acinetobacter	0.00000996	0.000722796	0.0000739	0.0000210	0.0052
	Rhizobium	0	0.000181896	0.000406264	0.0000463	0.0067
	Burkholderia	0	0.000191469	0.000395712	0.0000210	0.0092
	Schlegelella	0	0.000157962	0.000263808	0.00000842	0.0045
	Novosphingobium	0.00000996	0.000268057	0.000116075	0.0000210	0.0134
	Enhydrobacter	0	0.000220189	0.0000844	0.0000253	0.0026
	Acetobacter	0	0.00029199	0.0000317	0	0.0011
	Candidatus. Hamiltonella	0.00000498	0	0.000337674	0	0.0086
	Sphingomonas	0	0.000143602	0.000142456	0	0.0023
	Cellvibrio	0	0.0000766	0.000147732	0.0000505	0.0014
	Stenotrophomonas	0	0.000114881	0.000168837	0	0.0012
	Brevundimonas	0.00000498	0.0000191	0.000242703	0.0000168	0.0224
	Photobacterium	0	0.000253697	0.00000528	0	0.0168
	Zea	0.00000498	0.000105308	0.000121352	0	0.0264
	Janthinobacterium	0	0.000201043	0.00000528	0.00000421	0.0157
	Tepidimonas	0	0.000201043	0	0.00000421	0.0294
	Steroidobacter	0.00000498	0.0000622	0.000142456	0.00000421	0.0304
	Achromobacter	0	0.0000191	0.000195218	0.00000421	0.0185

Supplementary Table S5

Genera with significantly different abundances in each month from October 2015 to January 2016 of *Dendroctonus armandi* larvae of Actinobacteria, Firmicutes, Bacteroidetes, Acidobacteria, Tenericutes and Deinococcus-Thermus.

Phylum	Genus	mean abundance				*p* value
		**Oct. 2015**	**Nov. 2015**	**Dec. 2015**	**Jan. 2016**	
Actinobacteria	*Corynebacterium*	0.000164315	0.056023857	0.000416816	0.000340899	0.0251
	Knoellia	0.0000647	0.016533356	0.003529747	0.0000463	0.0162
	Blastococcus	0.0000797	0.001005213	0.008763692	0.000155719	0.0145
	Rhodococcus	0.00000996	0.005523883	0.001567018	0.000235683	0.0206
	Euzebya	0.0000149	0.002479525	0.000664795	0.0000126	0.0113
	Salinibacterium	0.0000149	0.001148815	0.0000580	0	0.0195
	Actinopolymorpha	0	0.001120094	0	0	0.0023
	Gordonia	0.0000199	0.000416445	0.000622586	0.00000842	0.0244
	Mycobacterium	0.0000349	0.000459526	0.0000739	0.00000421	0.0178
	Patulibacter	0	0.000186682	0.000358778	0	0.0069
	Micrococcus	0	0.000296777	0.000200494	0.00000421	0.0112
	Streptomyces	0	0.0000191	0.000337674	0	0.0074
	Propionibacterium	0.0000149	0.000201043	0.0000475	0.00000421	0.0123
	Pimelobacter	0	0.000205829	0.0000580	0	0.0046
	Dietzia	0	0.0000862	0.000147732	0.0000168	0.0227
Firmicutes	*Alloiococcus*	0.000527799	0.074251715	0.007365511	0.000425072	0.0240
	Anoxybacillus	0.00025892	0.058565609	0.007787603	0.000441906	0.0155
	Clostridium	0.000358505	0.001627487	0.04026233	0.000374568	0.0197
	Ammoniphilus	0.0000249	0.002154027	0.004669396	0.0000253	0.0146
	Solibacillus	0.0000199	0.001756729	0.00226347	0.0000673	0.0246
	Paenibacillus	0	0.00000957	0.002020767	0	0.0153
	Enterococcus	0.0000498	0.000531327	0.000216322	0.0000253	0.0211
	Staphylococcus	0.00000996	0.000339858	0.0000791	0	0.0056
	Aerococcus	0	0.000311137	0.0000791	0	0.0161
	Desemzia	0	0.000124455	0.000258532	0	0.0043
	Streptococcus	0	0.0000814	0.000221598	0	0.0165
	Bacillus	0	0.000114881	0.00013718	0	0.0034
	Lactococcus	0	0.000124455	0.0000950	0.00000421	0.0374
Bacteroidetes	*Chryseobacterium*	0.0000249	0.000627061	0.002701391	0.00012205	0.0182
	Sphingobacterium	0	0.000871184	0.000126628	0	0.0079
	Chitinophaga	0.00000498	0.0000574	0.000949708	0.00000842	0.0162
	Flavobacterium	0.000104564	0.000229763	0.000485406	0.0000210	0.0214
Acidobacteria	*Edaphobacter*	0.0000249	0.001900331	0.004674672	0.0000800	0.0182
Tenericutes	UNCLASSFIED	0.000607467	0.039820785	0.010531203	0.000227266	0.0157
Deinococcus-Thermus	*Meiothermus*	0.00000498	0.00000957	0.000759766	0	0.0072
